# Alcohol, Antidepressants, and Circadian Rhythms: Human and Animal Models

**Published:** 2001

**Authors:** Alan M. Rosenwasser

**Affiliations:** Alan M. Rosenwasser, Ph.D., is a professor in the Department of Psychology at the University of Maine, Orono, Maine

**Keywords:** circadian rhythm, antidepressants, AOD (alcohol or other drug) use pattern, physiological AODE (effects of AOD use, abuse, and dependence), hypothalamus, CNS (central nervous system) nuclei, brain pathway, serotonin, human study, animal study

## Abstract

Alcohol consumption (both acute and chronic) and alcohol withdrawal have a variety of chronobiological effects in humans and other animals. These effects are widespread, altering the circadian rhythms of numerous physiological, endocrine, and behavioral functions. Thus, some of alcohol’s negative health consequences may be related to a disruption of normal physiological timing. Most studies of alcohol’s chronobiological effects have been conducted under natural conditions in which environmental stimuli, such as regular cycles of light and darkness, act to coordinate circadian rhythms with the environment and with each other. However, such studies cannot distinguish between effects occurring directly on the circadian pacemaker and those occurring “downstream” from the pacemaker on the physiological control systems. Studies using animals have enabled researchers to begin to examine the effects of alcohol on circadian rhythms under so-called free-running conditions in experimental isolation from potential environmental synchronizers. These studies have provided preliminary evidence that alcohol’s chronobiological effects are indeed the result of direct influences on the circadian pacemaker itself. Furthermore, the effects of alcohol on animal circadian rhythms appear similar to the effects seen during administration of antidepressant drugs. Taken together with evidence that the chronobiological effects of alcohol withdrawal in human alcoholics are reminiscent of those described in depressed patients, these observations suggest that alcohol may produce antidepressantlike effects on the circadian pacemaker. One theory suggests that the effects of alcohol on the circadian pacemaker are mediated in part by alterations in serotonin, an important chemical involved in cellular communication within the circadian system. However, other neurochemical systems also are likely to be involved.

As evidenced by the articles assembled for this issue of *Alcohol Research & Health*, at least three separate lines of evidence suggest a link between alcohol ingestion and the regulation of the body’s daily biological, or circadian, rhythms regulating sleep and activity, body temperature, hormone secretions, and essentially all other important physiological and behavioral processes.

First, circadian rhythms modulate several behavioral and physiological responses to alcohol in both humans and experimental animals. Circadian modulation of a drug’s effect is not specific to alcohol, however, because circadian variations in drug effectiveness also are seen for numerous other substances, including other psychoactive drugs.

Second, the propensity for voluntary alcohol intake is influenced by the circadian system in both humans and animals. People and other animals with access to alcohol in studies essentially tend to drink at specific times of the day or night. For example, rats and mice exhibit maximal levels of voluntary alcohol consumption during the night phase of their circadian cycle. Because the animals’ daily pattern of alcohol intake closely resembles their normal daily patterns of food and water intake, the circadian modulation of alcohol intake may not reflect a specific influence on alcohol-seeking behavior, but may instead reflect an influence on active behavior in general.

Third, the normal circadian patterns of a variety of behavioral and physiological parameters (e.g., sleep and activity, body temperature, hormone secretions) are disrupted by alcohol administration, ingestion, and/or withdrawal. Such effects are the focus of this article, which examines alcohol’s effects on circadian rhythmicity in people and animals and explores some possible underlying neuropharmacological mechanisms. In addition, the effects of alcohol on circadian rhythms are compared with the chronobiological effects of antidepressants and with alterations in circadian rhythms seen in both human depression and in animal models of depression.

## Methodological Considerations in Chronobiology

In its simplest configuration, the circadian system can be conceived as comprising a central circadian clock, or pacemaker; a set of input pathways mediating the effects of various environmental synchronizers (such as light and darkness) on the pacemaker; and a set of output pathways conveying pacemaker signals to other regulatory systems of the brain and body. A stimulus or treatment that affects any one of these components may alter the expression of circadian rhythms. Furthermore, even stimuli that bypass the circadian system entirely, and that instead act directly on physiological control systems, can modify the overt expression of circadian rhythms.

Circadian biologists generally are interested in distinguishing between those effects mediated “upstream” on the circadian pacemaker and/or its input pathways and those mediated “downstream” on circadian output pathways and/or physiological control systems. In the chronobiological literature, stimuli that alter circadian rhythm expression via downstream mechanisms are said to produce “masking” of circadian rhythms. As implied by the term masking, such effects are generally considered to obscure the behavior of the underlying circadian pacemaker.

Given the complexity of the pathways influencing the body’s circadian rhythms, how can the mechanisms underlying the chronobiological effects of a drug or other stimulus be determined? Assume, for example, that alcohol administration alters the normal daily pattern of secretion of a particular hormone. Several different types of alterations are possible, including changes in the overall level (i.e., amount) of hormone secretion, the time of day at which the highest peak (or lowest trough) of secretion occurs, or the pattern of secretion over the course of the day and night.

Certainly, such findings *could* reflect effects of alcohol on an upstream circadian process; for example, on the range of fluctuation (referred to as the amplitude), the timing (referred to as the phase), or other parameters of the underlying circadian pacemaker itself. It also is possible, however, that these effects are mediated downstream from the circadian pacemaker—for example, on the physiological regulation of hormone synthesis, secretion, or degradation. In such a scenario, the circadian pacemaker could still be functioning normally, despite the apparent alteration in circadian rhythmicity. For these reasons, changes in the expressed daily rhythmic pattern cannot be assumed to reflect altered circadian clock function. Especially under normal unconstrained conditions, such as those prevailing in the subject’s typical environment, the expressed daily pattern of any behavioral or physiological rhythm reflects both the output of the underlying circadian pacemaker and a variety of pacemaker-independent masking effects.

The most direct way then to separate direct effects on the pacemaker from downstream masking effects is by using artificial experimental conditions in which subjects are isolated from normal periodic signals from the environment, such as the regular daily alternation of light and darkness. Under such conditions, circadian rhythms display periods (i.e., cycle lengths) that deviate somewhat from the strict 24-hour periods seen under normally synchronized conditions. This period, which is typically somewhat longer or shorter than 24 hours, is referred to as the “free-running” circadian period. Unlike the level, timing, or daily pattern of a synchronized rhythm, which reflects both circadian and masking effects as described previously, the free-running circadian period is considered to directly reflect the period of the underlying circadian pacemaker. Thus, experimental isolation from periodic factors in the environment— so-called free-running conditions—best allow for the separation of chronobiological effects resulting from perturbation of the circadian pacemaker and those resulting from perturbation of downstream processes.

In animal experiments, free-running circadian rhythms are expressed readily and measured easily under conditions of either constant light or constant darkness, as appropriate to the species under study. An extensive body of evidence indicates that the free-running circadian period—and thus the underlying circadian pacemaker—is influenced by a variety of environmental factors (especially light intensity but also temperature and food availability) as well as by certain organismal variables (e.g., endocrine status, behavioral activity, sleep, and possibly personality characteristics). Most relevant to the present discussion, the free-running circadian period also is modified by several pharmacological agents, including antidepressants, other psychoactive drugs and, as discussed later, alcohol.

Despite the obvious practical constraints and limitations, free-running circadian rhythm experiments also have been conducted using socially isolated and environmentally restricted human subjects. However, to the author’s knowledge, such methods have not been employed specifically to test potential pharmacological effects on the human circadian system. As an alternative to such restrictive experiments, researchers in human chronobiology also have developed methodological tools to help distinguish between pacemaker-dependent and pacemaker-independent effects on circadian rhythms, even without the need for prolonged free-running experiments. For example, under the so-called constant routine protocol, subjects are kept under conditions of continuous dim light, bed rest, supine posture, and sleep deprivation for 1 to 2 days to reveal the underlying phase and amplitude of the circadian pacemaker in the absence of known sources of masking. In addition, a variety of statistical tools have been developed in an attempt to statistically “purify” circadian rhythm data collected under even less constrained conditions by mathematically removing the effects of masking. Unfortunately, these procedures have not yet been applied to the study of alcohol and drug effects on human circadian rhythms. Thus, it must be emphasized that the effects of alcohol administration and withdrawal on human circadian rhythms reported to date do not provide conclusive evidence for effects of alcohol on the circadian pacemaker.

## Effects of Alcohol on Human Circadian Rhythms

Most studies of alcohol’s effects on human circadian rhythms have been conducted in chronic alcoholics undergoing alcohol abstinence and associated withdrawal. In contrast, much less is known concerning the circadian effects of alcohol in nonalcoholics or social drinkers. In one early study of nonalcoholic subjects (Mullin et al. 1933), evening alcohol consumption was reported to elevate subsequent nocturnal body temperature and to shift the overnight minimum in body temperature to an earlier time (i.e., a phase advance). In a more recent but limited study (*n* = 3), nighttime alcohol intake also elevated nocturnal body temperature, but produced a variety of complex effects on the circadian pattern, including an apparent shift in the temperature rhythm to a *later* time period (i.e., a phase delay) in one person (Eastman et al. 1994).

Several studies of abstinent alcoholics during acute and/or longer term alcohol withdrawal have reported abnormalities in the amplitude, timing, and/or patterning of circadian rhythms. Typically, such studies have used normal control subjects for comparison groups and have not examined circadian rhythms in alcoholics during maintained alcohol intake prior to withdrawal. Such studies cannot easily distinguish between effects occurring during chronic alcohol consumption (and persisting during abstinence) with those that may be triggered by withdrawal itself. Nevertheless, the reported effects on circadian organization generally are correlated with the severity of withdrawal symptoms (e.g., Sano et al. 1994) and may persist for several days or months before resolving fully.

Reported withdrawal-associated effects on circadian rhythms include phase-advances (i.e., earlier timing) of circadian rhythms in body temperature (Kodama et al. 1988), rapid-eye-movement (REM) sleep (Imatoh et al. 1986), and levels of 5-hydroxyindoleacetic acid (5–HIAA, the primary metabolic by-product of serotonin, an important chemical involved in communication among nerve cells) (Sano et al. 1993, 1994). In contrast, however, phase delays (i.e., later timing) have been described for circadian rhythms in blood cortisol, a key stress hormone (Iranmanesh et al. 1989). In addition, other studies have found that circadian patterns of both cortisol and melatonin (a hormone involved in sleep and circadian rhythm regulation) may be severely blunted or even completely abolished during alcohol withdrawal (Fonzi et al. 1994; Mukai et al. 1998; Schmitz et al. 1996). Finally, the overall levels of body temperature and cortisol secretion are elevated in alcohol withdrawal.

It is of considerable interest that a similar constellation of circadian rhythm alterations—including widespread changes in the phase, amplitude, and/or level of REM sleep, body temperature, cortisol, and melatonin rhythms—also have been described in depressed patients (Duncan 1996; Rosenwasser and Wirz-Justice 1997). The possible correspondence between clinical depression and alcohol withdrawal is further highlighted by findings that withdrawal-induced circadian phase-advances and temperature elevations were more dramatic in depressive than in nondepressive alcoholics (Kodama et al. 1988), implying a possible additive or interactive effect of these two states.

### Depression, Antidepressants, and Human Circadian Rhythms

Like the studies of circadian rhythms during alcohol withdrawal described above, the majority of circadian studies in depressed patients also have examined normally synchronized, rather than free-running, circadian rhythms. As emphasized earlier in this article, such studies cannot effectively separate effects on the circadian pacemaker from effects mediated downstream from the pacemaker. Unlike the literature on withdrawing alcoholics, however, limited data *are* available from depressed subjects studied during isolation from daily environmental time cues or under constant routine protocols. The results of these studies generally support the view that depression-related circadian rhythm abnormalities reflect, at least in part, changes in the functioning of the circadian pacemaker itself (Duncan 1996; Rosenwasser and Wirz-Justice 1997). For example, a few depressed patients studied under isolation conditions have shown preliminary evidence for unusually short free-running circadian periods, and depressed patients may express abnormalities in their circadian phase even when studied under constant routine protocols that control for possible masking effects. Furthermore, antidepressant drugs—including tricyclics, selective serotonin reuptake inhibitors (SSRIs), and monoamine oxidase inhibitors (MAOIs)—tend to reverse circadian abnormalities when administered to depressed patients (Duncan 1996).

Several hypotheses have been proposed suggesting that abnormal circadian rhythms may be a causal factor in depression and that the clinical efficacy of antidepressants may be partially mediated by their ability to normalize those abnormal circadian rhythms (Duncan 1996; Rosenwasser and Wirz-Justice 1997). Nevertheless, specific cause-and-effect mechanisms linking circadian rhythms and depression remain to be elucidated; possibly some physiological or behavioral correlate of depression is the cause of altered rhythmicity, rather than the other way around.

## Summary of Human Studies

Alterations in circadian rhythms are induced by acute alcohol administration, alcohol withdrawal, and antidepressant drugs, but the general lack of free-running experiments precludes any strong conclusions concerning possible effects of these treatments on the human circadian pacemaker. Nevertheless, the similarity of effects seen in depressed patients and in alcoholics undergoing withdrawal is consistent with the hypothesis that alcohol withdrawal produces a depressionlike affective state, and that alcohol produces antidepressantlike effects on the circadian system. To test this hypothesis explicitly, it will be necessary to conduct additional studies of both alcohol- and depression-related effects on circadian rhythms under free-running conditions or in constant routine protocols to better separate effects on the circadian pacemaker from downstream masking effects. Additional research using animals also will yield important information, as detailed in the following section.

### Effects of Alcohol on Animal Circadian Rhythms

In laboratory rats and mice, as in human subjects, alcohol administration alters the expression of circadian rhythms in a variety of behavioral, physiological, and endocrine functions. And as in the human literature discussed earlier, many of the animal experiments were conducted during the synchronization to periodic environmental signals, particularly to 24-hour light-dark cycles. These conditions mimic the human experience and may thereby increase the validity of animal findings if the primary aim is to extrapolate such findings to human behavior. From a chronobiological standpoint, however, such studies fail to take advantage of a major opportunity inherent in animal circadian rhythm research; that is, the ability to study free-running circadian rhythms under conditions of environmental isolation from potential synchronizers.

Under standard light-dark conditions, alcohol administration and/or voluntary alcohol ingestion have been reported to alter the phase, blunt the amplitude, or abolish the expression of circadian rhythms in a variety of behavioral and physiological functions, including locomotor activity, body temperature (Baird et al. 1998), sleep (Ehlers and Slawecki 2000; Rouhani et al. 1990), food intake (Barr 1988; Goldstein and Kakihana 1977), secretion of the stress-related hormone, corticosterone (Kakihana and Moore 1976), and other functions (El-Mas and Abdel-Rahman 2000; Rajakrishnan et al. 1999).

In addition, alcohol increases the overall level of corticosterone release and REM sleep and decreases body temperature. As a whole, these effects are remarkably similar to those described earlier for abstinent human alcoholics studied under synchronized, non-free-running conditions.

To date, only four reported studies have examined the effects of chronic alcohol treatment on free-running circadian rhythms, and two of these have been presented only in preliminary form.

Syrian hamsters were used in three of the studies. This animal is a popular model species in circadian rhythm research because of the remarkable precision of the hamster circadian pacemaker, especially as expressed in running-wheel activity. Thus, free-running circadian activity rhythms in the hamster show little day-to-day variability, allowing the free-running period to be measured with a high degree of accuracy. All three hamster studies showed small but reliable lengthening of the free-running period during exposure to 20 to 28 percent alcohol solutions (Joy and Turek 1989; Mistlberger and Nadeau 1992; Zucker et al. 1976). After terminating alcohol treatment, individual hamsters showed varying responses, including circadian period shortening, further period lengthening, and no period change (Mistlberger and Nadeau 1992).

**Figure 1:**
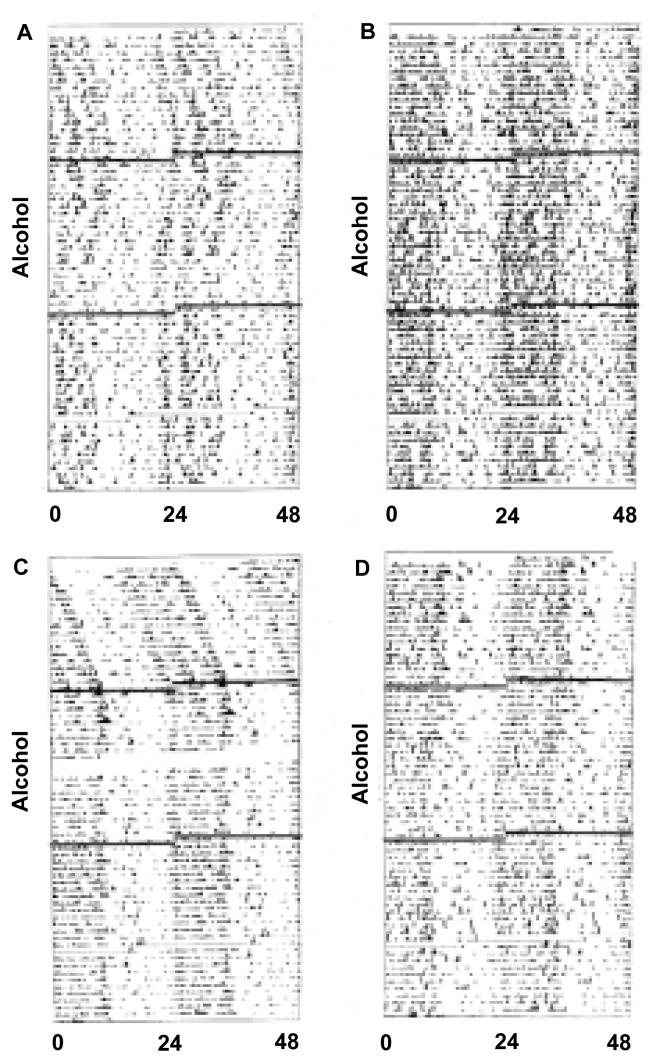
Panels show the circadian water-intake rhythms in four rats before, during, and after free-choice access to both a 10-percent alcohol solution and water. Time of day (a 48-hour span) is indicated along the horizontal axis, and successive days of the experiment are represented from top to bottom along the vertical axis (as shown by the breaks in the lines). The period of time the rats had access to alcohol is indicated by the label along the vertical axis. Dark areas in the charts show the times when the rats had the highest drinking activity. The animals were maintained in constant darkness to allow for the expression of free-running circadian rhythms—that is, in experimental isolation from any potential environmental synchronizer. Note that all four rats showed shortening of a free-running circadian period during the period of alcohol access as indicated by the relative decrease in rightward drift and/or increase in leftward drift of the times of highest activity during the alcohol access.

Our laboratory reported the only available data to date on the effects of alcohol treatment on free-running circadian rhythms in rats (Dwyer and Rosenwasser 1998). In that experiment, which was primarily designed to examine the effects of antidepressant treatment on circadian rhythms (see following section), adult rats that were treated with the antidepressant clomipramine or with saline soon after birth were offered a free choice between a 10-percent alcohol solution and plain water as adults. In apparent contrast to the findings with hamsters described earlier, the rats in both treatment groups consistently showed a shortening of the free-running circadian period during alcohol availability (see figure 1). Once alcohol treatment was stopped, the rats also exhibited a partial reversal of this effect.

Alcohol treatment thus appears to affect the circadian pacemaker in both rats and hamsters, but the effects of alcohol treatment on the free-running period was opposite in the two species. One possibility is that this apparent difference is related to differences in the species’ alcohol intake and/or metabolism. In comparison to typical laboratory rats and mice, Syrian hamsters exhibit much higher levels of spontaneous alcohol consumption, and greater preference for high-concentration alcohol solutions, with little evidence for an alcohol-withdrawal response. These behavioral observations are apparently linked to hamsters’ higher efficiency of alcohol metabolism relative to other species (Kulkosky and Cornell 1979; Piercy and Myers 1995). Nevertheless, additional studies will be required to explain why the two species showed different effects on the free-running period.

## Antidepressants, Animal Depression Models, and Circadian Rhythms

In contrast to the limited studies on alcohol effects, a more extensive animal research literature has examined the effects of antidepressant drugs and of established animal models of depression on circadian rhythms under both environmentally synchronized conditions (Gorka et al. 1996; Greco et al. 1990; Klemfuss and Kripke 1994; Yannielli et al. 1999) as well as free-running conditions (Rosenwasser and Wirz-Justice 1997). The effects of antidepressants on the free-running circadian period indicate that these drugs affect the circadian pacemaker. Although these effects have not been entirely consistent across studies (Klemfuss and Kripke 1994), period shortening has been described for desipramine, moclobemide, and fluoxetine; and period lengthening has been described for imipramine, clorgyline, and the antimanic agent lithium (Duncan 1996; Rosenwasser 1992; Rosenwasser and Wirz-Justice 1997). As described previously in regard to alcohol, anti-depressant-induced alterations in a free-running circadian period may persist or even be exacerbated after the drug has been terminated (Wollnik 1992). In addition, alterations in the free-running period also have been seen during treatment with other mood-altering drugs, including clonidine, an antihypertensive and putative depressogenic agent (Dwyer and Rosenwasser 2000; Rosenwasser 1996), and methamphetamine (Honma et al. 1991). Like antidepressants, these drugs also primarily affect the monoamine neurotransmitter systems, especially the serotonin and norepinephrine systems, known to be critically involved in the regulation of mood, arousal, and behavior.

Alterations in circadian rhythms also have been observed in several animal models of depression (Rosenwasser and Wirz-Justice 1997). Similar to the results just described for antidepressant drugs, both shortening or lengthening of the circadian period have been reported in different animal models characterized by depressionlike behavior. For example, lengthening of the free-running circadian period has been reported after repeated exposure to stressors and following olfactory bulb removal (a procedure that reduces levels of monoamine neurotransmitters), whereas period-shortening has been reported in genetically selected rat strains showing altered responsiveness to drugs affecting monoamine and acetylcholine neurotransmitter systems (Rosenwasser 1992; Rosenwasser and Wirz-Justice 1997).

Research from several laboratories has established that treatment with antidepressants early in life in otherwise normal rats produces behavioral and physiological effects in adulthood that resemble human depression. After neonatal treatment with antidepressants, such as clomipramine and desipramine, adult rats show alterations in sleep, sexual activity, and other behaviors that appear to mimic those seen in depressed patients. Of particular interest here are studies indicating that neonatal antidepressant treatment increases voluntary alcohol intake and decreases activity in the serotonin neurotransmitter system— findings that are parallel to observations in human subjects linking decreases in brain serotonin activity to both depression and alcohol consumption.

Four separate studies have examined free-running circadian rhythms in adult animals treated with antidepressants in early postnatal life; two of these studies used clomipramine-treated hamsters, the third one studied clomipramine-treated rats, and the fourth study used desipramine-treated rats. Although one hamster study failed to detect any significant effects of neonatal clomipramine treatment on circadian rhythms (Klemfuss and Gillin 1998), the other reported shortening of the free-running period (under constant light) and increased circadian amplitude (Yannielli et al. 1998). In rats, the researchers reported lengthening of the free-running period (in constant darkness) after neonatal desipramine treatment (Rosenwasser and Hayes 1994) and increased circadian amplitude and voluntary alcohol intake after both neonatal desipramine and clomipramine treatments (alcohol intake was not assessed in the hamster experiments) (Dwyer and Rosenwasser 1998; Rosenwasser and Hayes 1994). These studies indicate that neonatal antidepressant treatment, like other animal models of depression, is associated with alterations in the circadian pacemaker.

## Summary of Animal Studies

Similar to the human studies discussed earlier in this article, controlled animal experiments conducted under normally synchronized conditions (i.e., in the presence of a regular daily light-dark cycle) have demonstrated that alcohol and antidepressant drug treatments can affect the phase, amplitude, and patterning of circadian rhythms in a variety of measures. Of course, such studies are subject to the same analytical constraints described previously for human studies conducted under non-free-running conditions—specifically, an inability to distinguish effects on the circadian pacemaker from those mediated downstream from the pacemaker. In addition, however, several researchers have taken advantage of the greater potential for experimental control afforded by animal experiments to examine the effects of alcohol, antidepressants, and various animal depression models on free-running circadian rhythms. Such studies have identified unambiguous effects of these treatments on the circadian pacemaker, and suggest that the effects seen under normally synchronized conditions are at least partially the result of alterations in pacemaker function.

### Possible Cellular Bases for Alcohol Effects on the Circadian Pacemaker

In relatively simple organisms, such as plants and invertebrate animals, alcohol treatment alters the free-running circadian period and phase, demonstrating unambiguous effects on the circadian pacemaker (Bunning 1973; Bunning and Moser 1973; Cornelius and Rensing 1982). Indeed, similar effects have been seen for several other chemicals that—like alcohol—alter fundamental biophysical or biochemical cellular-level processes, including changes in the cell membrane and in protein synthesis. Many of these studies were conducted in an attempt to identify the basic cellular mechanisms responsible for circadian rhythmicity. Recently, the main timekeeping center of the body (i.e., the core oscillatory mechanism) was described at the molecular level as comprising a genetic feedback loop in which the proteins produced by specific circadian clock genes feed back to inhibit their own synthesis. A similar molecular feed-back loop appears to operate in both simple, one-cell organisms and within the specialized circadian pacemaker cells of complex animals (Dunlap 1999). Thus, at a cellular level, the effects of alcohol on the circadian pacemaker could be mediated by either (1) nonspecific effects on basic and widespread cellular processes or (2) specific effects on the expression of particular circadian clock-genes or clock-gene products.

### Possible Neuropharmacological Bases for Alcohol Effects on the Circadian System

In animals with complex nervous systems, the basic molecular oscillator described previously is contained within thousands of individual circadian pacemaker nerve cells (i.e., neurons). Normally, circadian rhythm-generating processes are synchronized among this population of neurons by one or more mechanisms of intercellular communication. Thus, at the nerve-cell-systems level of analysis, the effects of alcohol on the circadian pacemaker could be mediated by pharmacological effects on particular neurotransmitter or neuromodulator systems that mediate intercellular communication.

**Figure 2:**
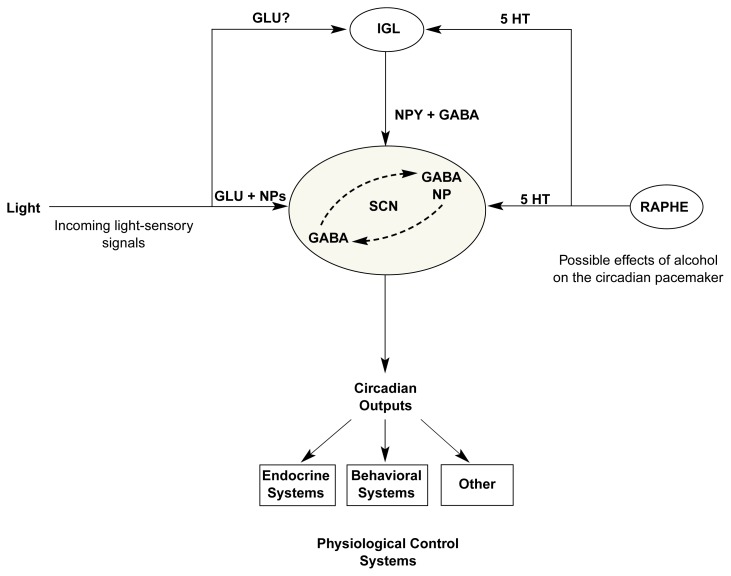
Schematic representation of the neural circuitry underlying the control of circadian rhythms. The circadian pacemaker resides just above the base of the brain in a distinct region of the hypothalamus called the suprachiasmatic nucleus (SCN). Cells of the SCN mutually interact and contain a number of chemicals important in cellular communication, including the neurotransmitter gamma-aminobutyric acid (GABA) and one or more neuropeptides (NP). The SCN receives incoming information from three primary sources: (1) the retina of the eye, which sends signals about environmental lighting to the SCN along a direct pathway that uses glutamate (GLU) as its primary transmitter, along with at least two different NPs; (2) the intergeniculate leaflet (IGL) of the visual thalamus, which receives retinal signals and sends signals to the SCN via a pathway that employs both GABA and neuropeptide Y (NPY) as transmitters; and (3) the raphe cell groups of the midbrain, which use the monoamine serotonin (5 HT) as the primary neurotransmitter and also send signals to the IGL. As indicated, the serotonin pathways to the SCN (and IGL) are most likely involved in mediating, at least in part, the effects of alcohol on the circadian pacemaker in the SCN. However, alcohol also is known to interact with GLU, GABA, and NP signaling elsewhere in the brain. Thus, these systems also may be important for the circadian effects of alcohol.

The mammalian circadian pacemaker resides just above the base of the brain, in a distinct region of the hypothalamus called the suprachiasmatic nucleus (SCN) (Buijs et al. 1996). Individual neurons within the SCN are capable of generating self-contained autonomous circadian timing. However, these thousands of cellular oscillators typically remain in synchrony and thus generate the coherent output necessary for a pacemaker. Three major neural pathways relay information to the SCN (see figure 2):

- A direct pathway that emerges from a specialized subset of retinal neurons (i.e. retinal ganglion cells), separate from the retinal neurons that underlie visual perception. This pathway is referred to as the retinohypothalamic tract (RHT).
- A pathway that emerges from a specialized region in the thalamus called the intergeniculate leaflet (IGL). Although this region receives input from retinal ganglion cells, it also is separate from nearby regions that contribute to visual perception. The pathway from the IGL to the SCN is called the geniculohypothalamic tract (GHT).
- A pathway that emerges from the neurons of the raphe nuclei of the midbrain, known to be important in the regulation of mood, sleep, arousal, and other behavioral functions. This pathway transmits information not only to the SCN but also to the IGL.

Functionally, neural connections from the eye to the SCN and the IGL mediate the effects of light on the SCN circadian pacemaker, whereas pathways from the raphe to the SCN and IGL are thought to mediate, in part, the effects of certain nonphotic stimuli, including behavioral activity, sleep, and arousal on the SCN pacemaker. Thus, light-related and behavior-related signals converge and interact within the IGL and within the SCN itself.

These neural pathways transmit information by way of several chemical signals. The RHT releases the excitatory amino acid glutamate as its primary transmitter, which acts on several different subtypes of glutamate-docking molecules (i.e., receptors) within the SCN. In addition, the RHT releases other chemicals (i.e., the neuropeptides, substance P, and PACAP) that probably act along with glutamate as co-transmitters. Neurons of the GHT pathway release both the inhibitory amino acid transmitter gamma-aminobutyric acid (GABA) as well as a neuropeptide called neuropeptide Y (NPY). Neural pathways from the midbrain raphe to the SCN and IGL release the monoamine transmitter, serotonin, and other transmitters, probably including GABA. In addition to the signals released by these input pathways, intrinsic SCN neurons display diverse chemical characteristics. Although most or all SCN neurons appear to release GABA, individual SCN neurons also may release one or more of a wide variety of neuropeptides.

Several of the neurotransmitter systems just described are known to be modulated by alcohol, including serotonin, GABA, glutamate, and various neuropeptides. For example, acute alcohol consumption facilitates the effects of serotonin (by increasing transmitter release and/or by blocking transmitter reuptake), whereas chronic treatment with alcohol may lead to adaptative changes in serotonin receptors (LeMarquand et al. 1994).

Thus, the serotonin system is suppressed during alcohol withdrawal, as it is in depression. In view of the extensive animal research implicating a major role for serotonin in the regulation of the circadian pacemaker (Mistlberger et al. 2000; Morin 1999; Rea and Pickard 2000), this neurochemical similarity may be related to a general similarity between the circadian rhythm changes seen during alcohol withdrawal and in depressed patients. These observations suggest the hypothesis that alcohol-induced alterations in serotonin activity within the SCN and/or IGL are responsible in part for the effects of alcohol on the circadian pacemaker. From this perspective, the effects of alcohol on the circadian pacemaker may be viewed as similar to those seen with antidepressants and other mood-altering drugs that target the serotonin neurotransmitter system.

Nevertheless, other neurotransmitter systems may mediate the effects of alcohol on the circadian pacemaker. For example, acute alcohol administration facilitates the neural inhibitory effects of GABA, found within both SCN neurons and GHT, and inhibits the excitatory effects of glutamate, found within the RHT. Furthermore, chronic alcohol administration results in tolerance to these effects as a result of adaptive changes in GABA and glutamate receptors (Faingold et al. 1998). These receptor adaptations are thought to be responsible for the withdrawal syndrome that occurs during acute abstinence after chronic alcohol treatment.

Preliminary evidence also suggests that chronic alcohol administration may produce adaptive changes (e.g., a downregulation) in GABA receptors within the SCN circadian pacemaker, because this treatment blunts the effects of subsequent administration of the benzodiazepine triazolam on free-running circadian rhythms in hamsters (Joy and Turek 1989) (both alcohol and benzodiazepines act on a particular class of GABA receptor, the GABA_A_ receptor, to facilitate the neural inhibitory effects of GABA). On the other hand, this result should be replicated in light of reports that the specific molecular elements that confer alcohol sensitivity to the GABA_A_ receptor are poorly expressed in the SCN, relative to other brain regions (Madeira et al. 1997; Kawahara et al. 1993). Unfortunately, possible alterations in circadian pacemaker function resulting from alcohol’s action on the glutamate receptors have not been investigated, but given the well-defined role of glutamate in signaling light information to the SCN, such studies are clearly warranted.

Finally, alcohol consumption results in widespread reductions in the synthesis of several hypothalamic neuropeptides, including those within the SCN and IGL (Clark et al. 1998; Madeira et al. 1997; Madeira and Paula-Barbosa 1999), and this suppression of neuropeptide function also could underlie alcohol-induced alterations in circadian pacemaker function. It is interesting to note that alcohol-induced alterations in both SCN peptide expression (Madeira et al. 1997) and the free-running circadian period (Mistlberger and Nadeau 1992) may persist, or even be exacerbated, after discontinuation of alcohol treatment. Again, additional research will be necessary to determine whether changes in neuropeptide activity are partly responsible for the chronobiological effects of alcohol.

## Conclusion

Alcohol treatment and alcohol withdrawal produce a variety of effects on the expression of circadian rhythms in people and in experimental animals. Although much of this research has been conducted under normally synchronized conditions, experiments on free-running rhythms have revealed that the chronobiological effects of alcohol are at least partially the result of alcohol effects on the circadian pacemaker.

Alcohol is characterized by an exceptionally complex and widespread profile of pharmacological effects on the mammalian brain, and this complex profile provides several possible avenues for alcohol to affect the circadian pacemaker. As emphasized in this review, several lines of evidence implicate an important role for the neurotransmitter serotonin in these effects, including (1) the similarity between the chronobiological effects of alcohol and antidepressants on free-running rhythms in animals, (2) the similarity between the chronobiological effects of alcohol withdrawal and depression in humans, and (3) the extensive literature linking serotonin to depression, to alcohol intake, and to circadian rhythm regulation. Nevertheless, it is likely that the chronobiological effects of alcohol also are mediated in part by amino acid (e.g., GABA, glutamate) and neuropeptide transmission. Considerable additional research will be required to further elucidate the neurochemical effects of alcohol within the circadian pacemaker and to clarify the mechanisms mediating the effects of alcohol and other psychoactive drugs on circadian rhythms.
